# High-resolution mapping of traffic related air pollution with Google street view cars and incidence of cardiovascular events within neighborhoods in Oakland, CA

**DOI:** 10.1186/s12940-018-0382-1

**Published:** 2018-05-15

**Authors:** Stacey E. Alexeeff, Ananya Roy, Jun Shan, Xi Liu, Kyle Messier, Joshua S. Apte, Christopher Portier, Stephen Sidney, Stephen K. Van Den Eeden

**Affiliations:** 1 Division of Research, Kaiser Permanente Northern California, 2000 Broadway, Oakland, CA 94612 USA; 2 Environmental Defense Fund, New York, NY USA; 3 Dept. of Civil, Architectural and Environmental Engineering, University of Texas at Austin, Austin, TX USA

**Keywords:** Air pollution, Cardiovascular disease, Coronary heart disease, Mortality, Mobile monitoring

## Abstract

**Background:**

Some studies have linked long-term exposure to traffic related air pollutants (TRAP) with adverse cardiovascular health outcomes; however, previous studies have not linked highly variable concentrations of TRAP measured at street-level within neighborhoods to cardiovascular health outcomes.

**Methods:**

Long-term pollutant concentrations for nitrogen dioxide [NO_2_], nitric oxide [NO], and black carbon [BC] were obtained by street-level mobile monitoring on 30 m road segments and linked to residential addresses of 41,869 adults living in Oakland during 2010 to 2015. We fit Cox proportional hazard models to estimate the relationship between air pollution exposures and time to first cardiovascular event. Secondary analyses examined effect modification by diabetes and age.

**Results:**

Long-term pollutant concentrations [mean, (standard deviation; SD)] for NO_2_, NO and BC were 9.9 ppb (SD 3.8), 4.9 ppb (SD 3.8), and 0.36 μg/m3 (0.17) respectively. A one SD increase in NO_2_, NO and BC, was associated with a change in risk of a cardiovascular event of 3% (95% confidence interval [CI] -6% to 12%), 3% (95% CI -5% to 12%), and − 1% (95% CI -8% to 7%), respectively. Among the elderly (≥65 yrs), we found an increased risk of a cardiovascular event of 12% for NO_2_ (95% CI: 2%, 24%), 12% for NO (95% CI: 3%, 22%), and 7% for BC (95% CI: -3%, 17%) per one SD increase. We found no effect modification by diabetes.

**Conclusions:**

Street-level differences in long-term exposure to TRAP were associated with higher risk of cardiovascular events among the elderly, indicating that within-neighborhood differences in TRAP are important to cardiovascular health. Associations among the general population were consistent with results found in previous studies, though not statistically significant.

**Electronic supplementary material:**

The online version of this article (10.1186/s12940-018-0382-1) contains supplementary material, which is available to authorized users.

## Background

Long-term exposure to traffic-related air pollutant concentrations (TRAP) and to particulate matter less than 2.5 μm in diameter (PM_2.5_) have been associated with increased risk of cardiovascular disease (CVD) events in many epidemiological studies. A recent review and meta-analysis found that long-term exposure to traffic-related air pollutants (elemental carbon and nitrogen dioxide [NO_2_]) and to PM_2.5_ were associated with increased risk of all-cause mortality and cardiovascular mortality [1]. Long-term exposure to PM_2.5_ and to TRAP has also been associated with incidence of non-fatal CVD events [2, 3]. However, it is unclear whether associations between specific TRAP pollutants and CVD events are causal. The most recent US Environment Protection Agency Integrated Science Assessment for Oxides of Nitrogen (2016) determined that existing literature was “suggestive of, but not sufficient to infer, a causal relationship between long-term exposure to NO2 and cardiovascular health effects” [4]. Furthermore, few studies have examined the relationship of cardiovascular outcomes with nitric oxide (NO) separately from NO_2_ [5].

Traffic-related air pollution has high spatial variability within urban neighborhoods. A recent study of intra-urban spatial variability of TRAP within New York City found that concentrations ranged 3-fold for PM_2.5_, 5-fold for black carbon (BC) and over 10-fold for NO_2_ across 155 sites [6]. Thus, characterization of the health risk of spatially variable TRAP is vital to inform local decision making, such as zoning and transportation planning. Although land use regression techniques have improved the ability to characterize some of the spatial variability of pollutants over large areas, these methods are still limited in their ability to characterize the full distribution of highly spatially variable TRAP exposures within highly variable or idiosyncratic urban neighborhoods [7] and predictions are sensitive to variable selection [6, 8]. New research is emerging on the use of mobile monitoring to better characterize spatial variability of air pollutants without needing to carry out extensive modelling and prediction [9–11], but few studies have examined TRAP exposures estimated by mobile monitoring in relation to clinical health outcomes.

Building on the recent development of mobile platforms for air pollutant sensors with quick response time, greater portability and higher global positioning system resolution, we have carried out an intensive campaign to measure and map the spatial variation in NO, NO_2_ and BC levels on every street at 30 m resolution in three neighborhoods in Oakland [7]. The results of the air pollution measurement campaign highlighted the remarkable variation in pollutant concentrations, with 8-fold variation between street-level measurements over the study area [7]. To the best of our knowledge, no previous epidemiology study has linked cardiovascular health outcomes with measurements that capture this fine-scale variation in the NOx and BC components of TRAP. Using these high-resolution street-level measurements of NO, NO_2_ and BC, we conducted this study to better understand the effect of street to street variation in TRAP on incidence of cardiovascular events within neighborhoods.

## Methods

### Study cohort

Kaiser Permanente Northern California (KPNC) is a large integrated health care system that provides comprehensive medical services to 4 million members. This retrospective cohort study included 41,869 KPNC adult members who lived in the geographic study region within Oakland, CA during January 1, 2010 to December 31, 2015.Entry into the cohort required one full year of prior KPNC membership to ascertain existing chronic conditions before starting follow-up. Continuous KPNC membership (defined as gaps of no more than 90 days) and residency within the study region was required to remain in the cohort. Subjects with a history of prior CVD (myocardial infarction, coronary revascularization, stroke, or congestive heart failure) were excluded.

### Geographic study region

The geographic study region was defined by the three neighborhoods within Oakland, CA where mobile monitoring of TRAP was carried out [7]. The city of Oakland spans 202 km^2^ and includes urban, residential, and industrial areas, and also includes both flat and hilly terrain. TRAP measurements were carried out on streets in West Oakland (10.4 km^2^), Downtown Oakland (4.5 km^2^) and East Oakland (15.4 km^2^). Additional file 1: Figure S1 in the Supplementary Material illustrates the study region within Oakland, showing the neighborhoods where TRAP measurements were collected. These neighborhoods consisted of a variety of land uses including urban offices and residences in high rise buildings, commercial areas, industrial areas and warehouses, mixed land use, and low and high density residential areas. Finally, two major highways run through these neighborhoods (Additional file 1: Figure S1), exposing many streets and residences to high volumes of traffic.

### Cardiovascular events

Cardiovascular events were determined from electronic medical records based on ICD-9 and ICD-10 codes. The cardiovascular event outcomes for this study included myocardial infarction (MI; ICD-9 code 410.x, ICD-10 code I21.x to I22.x), coronary revascularization (ICD-9 code 36.x, ICD-10 procedure code 02.x), stroke (ICD-9 code 431.x to 434.x, and 436.0, ICD-10 code I60.x, I61.x, I63.x, and I64.x), death from coronary heart disease and death from cerebrovascular disease. Deaths of KPNC patients were collected from mortality files which combine KPNC data, California state death data, and Social Security Administration data to determine a patient’s vital status. The combinations of CVD event types were based on a previous study of long-term exposure to air pollution and incidence of cardiovascular events [2].

### Air pollution exposure

The spatial mapping of NO, NO_2_ and BC have been described in detail previously [7]. In brief, we partnered with Google Earth Outreach and outfitted two Google Street View cars (cars used by Google to take panoramic pictures of streets which are often featured in Google Maps and Google Earth), with fast-response analyzers and two independent GPS (Global Position System) receivers with ~ 3 m precision. NO was measured by chemiluminescence (Model CLD64, Eco Physics AG, Switzerland), NO_2_ was measured using 450 nm cavity-attenuation phase-shift spectroscopy (Model T500 U, Teledyne Inc., San Diego, CA), and black carbon (BC) particles were measured using a photoacoustic extinctiometer (Droplet Measurement Technologies, Boulder, CO). Drive plans for the cars were designed to carry out a seasonally balanced, repeated measurement campaign that covered different times of day (8 am to 6 pm) across the workweek for every road in the selected neighborhoods (West Oakland, Downtown and East Oakland; 30 km^2^ area). The measurement campaign collected an average of 31 days and 200 1-Hz measurements between May 2015 and May 2016 for each 30 m of road in the study design. The large resulting data set (3 × 10^6^ 1-Hz measurements; 24,000 total vehicle-km on 750 road-km) is unique with very high coverage density and repeat-visit frequency. Measurements were temporally corrected to a daily average using the ratio of the hourly average and the daily average from the West Oakland site of the Bay Air Quality Management District. For every 30 m road length, the median of all 1 Hz concentrations measured over the year was computed to provide a long term pollutant map of BC, NO and NO_2_. The median long-term exposure to each traffic-related air pollutant was chosen as the exposure of interest to be more robust to stochastic extreme concentrations (outliers). Exposures at 30 m resolution were used in the main analyses. Sensitivity analyses also considered averaged median exposures within a buffer of 60 m radius and 120 m radius. Each subjects’ address of residence at entry into the study cohort was geocoded and linked to the location of the closest air pollution measurement. Subjects were censored at the date of a change of address using the address history dates obtained from KPNC membership databases.

### Covariates

Model covariates were chosen a priori and included age, sex, race, body mass index (BMI), smoking status, comorbidities (diabetes, COPD, hypertension, hyperlipidemia), hypertensive medication use, statin medication use, and neighborhood socioeconomic status (SES) at the block-group level. Covariates were selected on the basis of plausibility and previously published relationships [2, 12, 13]. Individual-level covariates and pharmacy data were obtained from electronic medical records. Each subject’s address of residence was linked to block-group level Census data. We computed the neighborhood deprivation index (NDI) as a measure of neighborhood SES using census block group level variables of income/poverty, education, employment characteristics, housing and occupation [14].

### Statistical analysis

Cox proportional-hazards (PH) regression was used to model the time to the first cardiovascular event. Subjects were censored at the time of non-cardiovascular related death, disenrollment, at change of address, or at the end of follow-up on Dec 31, 2015, whichever occurred first. All covariates were included as baseline covariates in the regression models. We checked the proportional hazards assumption of the Cox model by assessing diagnostic plots and by testing for the statistical significance of interaction terms with time.

To model the shape of the association between each air pollutant and the incidence of cardiovascular disease, we first assessed the validity of assuming a linear relationship. We used restricted cubic splines to flexibly model the association between each air pollutant and the incidence of cardiovascular disease, and we formally tested for any evidence of a nonlinear relationship using a Wald test for the statistical significance of the spline terms. When no evidence of nonlinearity was found, we then included each air pollutant as a continuous measure and modeled its linear effect, estimating the hazard ratios associated with an increase of one standard deviation in the level of median long-term exposure.

Primary analyses examined the main effect of NO_2_, NO and BC independently on cardiovascular events and cardiovascular mortality in the full cohort. Secondary analyses examined the relationship of NO_2_, NO and BC with risk of cardiovascular outcomes in two potentially susceptible subgroups: elderly (age 65+) and diabetics. We used interaction terms to allow the estimated effects of TRAP to vary by group, and we tested for statistically significant differences in the estimated effects of TRAP by group.

We conducted several sensitivity analyses to better understand the impact of our modeling assumptions. One sensitivity analysis estimated the hazard ratios under an alternate specification of the Cox PH model using age as time scale with left censoring occurring at the age of inclusion. Another sensitivity analysis estimated the hazard ratios for alternate exposure calculations where median long-term TRAP exposure was calculated using a distance-weighted average within a buffer zone of 60 m and 120 m radius. A third sensitivity analysis examined whether the relationship of TRAP to risk of CVD events varied by neighborhood. For all statistical tests, a level of α = 0.05 was used to determine statistical significance. All analyses were conducted using SAS software, version 9.3 (SAS Institute).

## Results

The retrospective study cohort included 41,869 subjects who were followed for up to 6 years, with a total of 105,923 person-years of observation included in the analyses and an average follow-up time of 2.5 years per person. A total of 693 subjects (1.7%) had at least one cardiovascular event during follow-up.

The characteristics of the study cohort are given in Table 1. Our adult study cohort was predominantly middle aged or younger, with 57.0% ages 18 to 39, 35.1% ages 40 to 64, and 7.8% ages 65 and above. A majority of subjects were female (53.0%). The cohort was racially and ethnically diverse: African Americans comprised 38.4% of the study cohort, Caucasians comprised 24.0%, Hispanics 22.2%, and Asians 12.3%. The cohort members lived in neighborhoods that were more deprived (lower SES) than the neighborhoods of the average KPNC member: the study cohort had a mean NDI of 0.99, meaning that the average member of this study cohort lived in a neighborhood that had one standard deviation more deprivation than the average KPNC member since the NDI was standardized across the neighborhoods of all KP members to have mean zero and SD one. Similarly, the census block-group median household income was $37,600 (Interquartile Range [IQR] $27,100 to $49,200).

**Table 1:** Descriptive characteristics of the study cohort at baseline

	Number	Percent
*Age*		
18 to 39	23,883	57.0
40 to 64	14,708	35.1
65+	3278	7.8
*Sex*		
Male	19,682	47.0
Female	22,187	53.0
*Race*		
African American	14,515	38.4
Caucasian	9068	24.0
Hispanic	8376	22.2
Asian	4634	12.3
Other	1170	3.1
Unknown	4106	
*BMI* ^a^		
Normal (18.5–24.9)	13,474	32.8
Underweight(< 18.5)	622	1.5
Overweight(25–29.9)	13,214	32.2
Obese(> = 30)	13,775	33.5
*Smoking* ^a^		
Never	26,774	67.7
Former	6148	15.5
Current	6647	16.8
*Co-morbidities*		
Diabetes	2898	6.9
COPD	4817	11.5
Hypertension	8069	19.3
Hyperlipidemia	6613	15.8
*Medication*		
Hypertensive medication	5639	13.5
Statin medication	2554	6.1
*Neighborhood SES*		
Neighborhood Deprivation Index*, Mean SD*	0.99	0.89
Census Household Annual Income in thousands, Median (IQR)	37.6	(27.1, 49.2)
*Region of Oakland*		
Downtown Oakland	12,608	30.1
East Oakland	19,226	45.9
West Oakland	10,035	24.0

^a^Includes only non-missing data

**Fig. 1:**
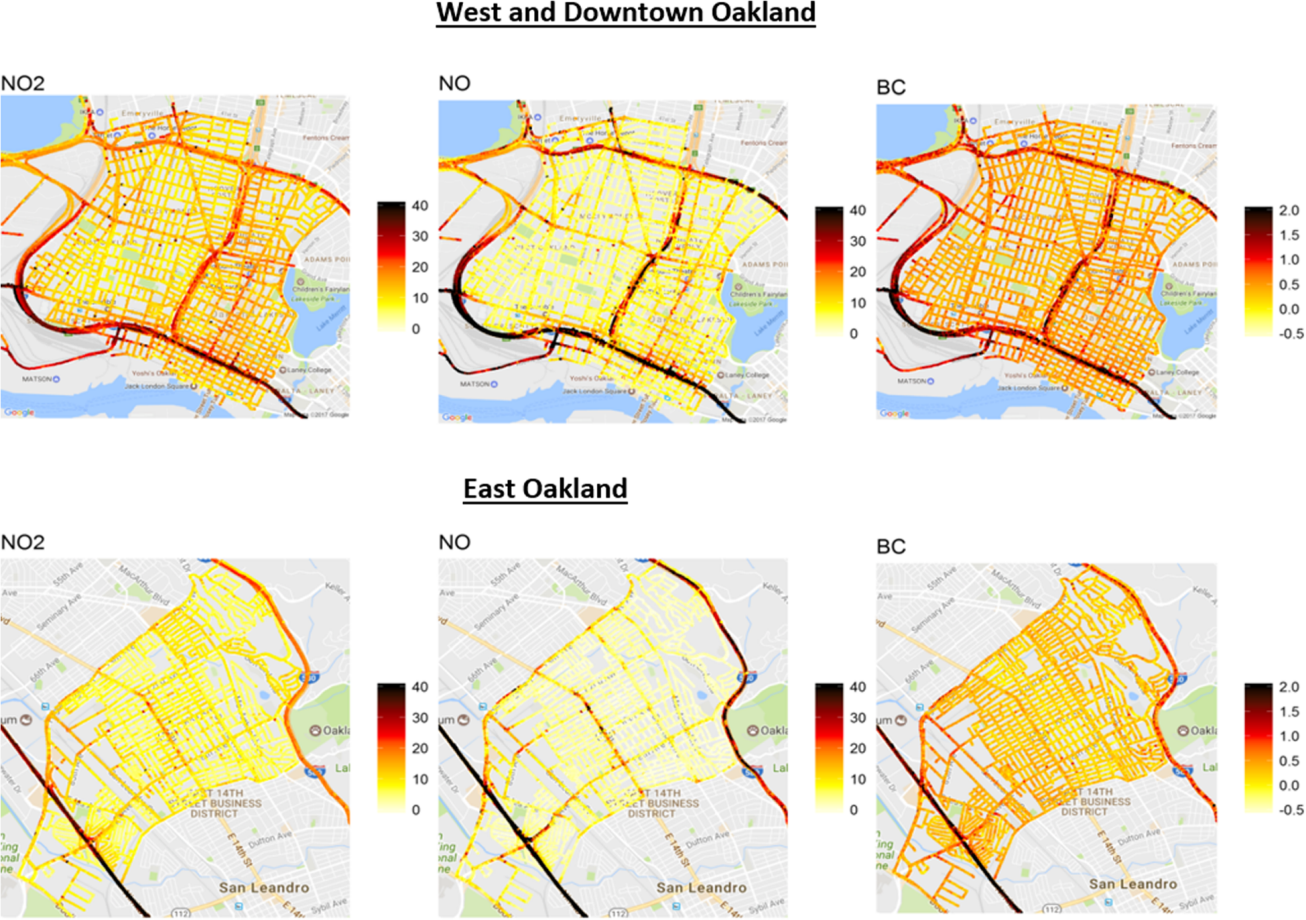
Median long-term street-level exposures to traffic-related air pollution in West/Downtown Oakland and in East Oakland

**Fig. 2:**
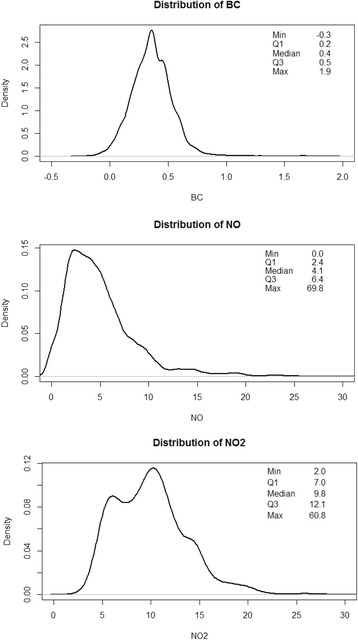
Distribution of exposures to traffic-related air pollution at study cohort residential address locations in Oakland

Median long-term air pollution exposures in Oakland at 30 m resolution are shown in Figure 1. Study cohort residential addresses had a mean exposure to NO_2_ of 9.9 ppb (standard deviation [SD] 3.8), a mean exposure to NO of 4.9 ppb (SD 3.8), and a mean exposure to BC of 0.36 μg/m^3^ (SD 0.17). Figure 2 illustrates the distribution of each TRAP exposure at study cohort residential address locations in Oakland. TRAP exposures at study cohort residential addresses were positively correlated with each other; NO_2_ and NO had the strongest correlation (*r* = 0.77) and black carbon was moderately correlated with NO_2_ (*r* = 0.58) and with NO (*r* = 0.59). Scatterplots of the bivariate relationships of pollutant pairs are shown in Additional file 1: Figure S2 (Supplementary Material). Area-level neighborhood socioeconomic measures were weakly negatively correlated with air pollution measures (correlations ranged from − 0.33 to − 0.04).

**Fig. 3:**
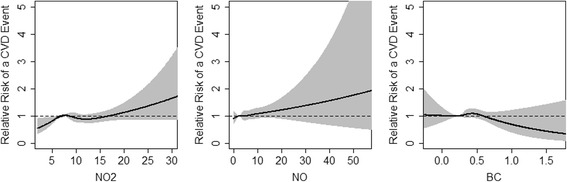
Estimated dose-response relationship between traffic-related air pollution exposure and relative risk of an incident cardiovascular event using cubic splines

Table 2 shows the estimated hazard ratios for the risk of an incident cardiovascular event associated with an increase of one standard deviation in the exposure to median long-term traffic-related air pollution, adjusted for covariates. An increase of one standard deviation in the exposure median long-term traffic-related air pollution was associated with an adjusted hazard ratio of 1.03 for NO_2_ (95% confidence interval [CI] 0.94 to 1.12), 1.03 for NO (95% CI 0.95 to 1.12), and 0.99 for BC (95% CI 0.92 to 1.07) for the time to the first cardiovascular event or death. The magnitude of effects observed was largest for the outcome of cerebrovascular disease death, 1.38 for NO_2_ (95% CI 0.93 to 2.06) and 1.13 for NO (95% CI 0.86 to1.49), and this outcome also had the fewest number of events (*N* = 27). In general, the associations of NO_2_ and NO with CVD outcomes were suggestive although none of the associations reached statistical significance. We found that our Cox PH models satisfied the proportional hazards assumption, which we verified by assessing diagnostic plots and by testing for the statistical significance of interaction terms with time. Figure 3 illustrates the relationship between the incidence of cardiovascular disease and each air pollutant when modeled using restricted cubic splines, where we found no statistically significant evidence of a nonlinear relationship. The estimated relative risk of an incident cardiovascular event associated with a change in the exposure to median TRAP exposure is shown relative to a reference level at the 25th percentile of exposure to each pollutant.

**Table 2:** Risk of an incident cardiovascular event associated with each air pollutant

Outcome	Events	Hazard Ratio (95% CI)^a^		
		NO_2_	NO	Black carbon
First cardiovascular event				
Cardiovascular event or death^b^	693	1.03 (0.94, 1.12)	1.03 (0.95, 1.12)	0.99 (0.92, 1.07)
Myocardial infarction	224	1.08 (0.93, 1.26)	1.08 (0.95, 1.23)	1.05 (0.91, 1.20)
Revascularization	130	0.99 (0.81, 1.22)	1.01 (0.84, 1.21)	1.03 (0.86, 1.23)
Stroke	325	0.97 (0.85, 1.11)	0.98 (0.87, 1.11)	0.96 (0.85, 1.08)
Coronary heart disease event or death^c^	394	1.05 (0.94, 1.18)	1.06 (0.95, 1.17)	1.05 (0.95, 1.16)
Cerebrovascular disease event or death^d^	337	0.99 (0.87, 1.12)	0.99 (0.88, 1.12)	0.95 (0.85, 1.07)
Cardiovascular death				
Coronary heart disease death	130	1.09 (0.89, 1.34)	1.05 (0.88, 1.25)	1.12 (0.94, 1.33)
Cerebrovascular disease death	27	1.38 (0.93, 2.06)	1.13 (0.86, 1.49)	0.92 (0.58, 1.45)

All models were adjusted for age, race, sex, BMI, NDI, smoking, baseline co-morbidities, medications

^a^Estimated hazard ratios per increase of one standard deviation in the median long-term exposure to each traffic-related air pollutant

^b^Events include myocardial infarction, coronary revascularization, stroke, death from coronary heart disease, and death from cerebrovascular disease

^c^Events include myocardial infarction, coronary revascularization, and death from coronary heart disease

^d^Events include stroke and death from cerebrovascular disease

Table 3 shows the association between exposure to median long-term traffic-related air pollution and the risk of an incident cardiovascular event among the elderly (age 65+) and among the non-elderly (age < 65). Among the elderly, an increase of one standard deviation in the exposure to median long-term traffic-related air pollution was associated with an adjusted hazard ratio of 1.12 for NO_2_ (95% CI 1.02 to 1.24), 1.12 for NO (95% CI 1.03 to 1.22), and 1.07 for BC (95% CI 0.97 to 1.17) for the time to the first cardiovascular event or death. This risk of any cardiovascular event or death represented a statistically significant difference from the risk among the non-elderly (*p*-values for interaction for NO_2_, NO and BC were < 0.001, 0.002, and 0.042 respectively). Effects of TRAP exposure on each cardiovascular events and death category generally appeared stronger among the elderly than among the non-elderly, and there were statistically significant differences in effects among the elderly as compared to the non-elderly for the risk of any cardiovascular event or death, myocardial infarction, and coronary heart disease event or death for all three TRAP exposures.

**Table 3:** For elderly and non-elderly, risk of an incident cardiovascular event associated with each air pollutant

Outcome	Events	Hazard Ratio (95% CI)^a^		
		NO_2_	NO	Black carbon
Elderly (age 65+; *N* = 3278):				
First cardiovascular event				
Cardiovascular event or death^b^	379	1.12 (1.02, 1.24)^§^	1.12 (1.03, 1.22)^§^	1.07 (0.97, 1.17)^§^
Myocardial infarction	121	1.20 (1.02, 1.42)^§^	1.20 (1.06, 1.36)^§^	1.15 (0.98, 1.36)^§^
Revascularization	54	1.04 (0.82, 1.33)	1.15 (0.94, 1.40)	1.00 (0.79, 1.27)
Stroke	172	1.03 (0.88, 1.19)	1.00 (0.86, 1.16)	0.99 (0.86, 1.15)
Coronary heart disease event or death^c^	226	1.16 (1.02, 1.31)^§^	1.17 (1.06, 1.29)^§^	1.15 (1.02, 1.29)^§^
Cerebrovascular disease event or death^d^	182	1.05 (0.91,1.21)	1.03 (0.89, 1.18)	0.98 (0.85, 1.14)
Cardiovascular death				
Coronary heart disease death	92	1.16 (0.93, 1.44)	1.11 (0.93, 1.33)	1.22 (1.01, 1.46)^§^
Cerebrovascular disease death	23	1.37 (0.90, 2.08)	1.13 (0.85, 1.50)	0.92 (0.57, 1.50)
Non-elderly (age ≤ 65; *N* = 38,591):				
First cardiovascular event				
Cardiovascular event or death^b^	314	0.93 (0.83, 1.03)^§^	0.94 (0.84, 1.05)^§^	0.92 (0.83, 1.02)^§^
Myocardial infarction	103	0.96 (0.80, 1.16)^§^	0.83 (0.65, 1.04)^§^	0.94 (0.80, 1.12)^§^
Revascularization	76	0.96 (0.77, 1.20)	0.86 (0.66, 1.12)	1.05 (0.86, 1.28)
Stroke	153	0.91 (0.77, 1.06)	0.95 (0.80, 1.12)	0.92 (0.80, 1.07)
Coronary heart disease event or death^c^	168	0.93 (0.80, 1.07)^§^	0.82 (0.69, 0.99)^§^	0.95 (0.83, 1.08)^§^
Cerebrovascular disease event or death^d^	155	0.91 (0.78, 1.07)	0.94 (0.79, 1.12)	0.92 (0.80, 1.06)
Cardiovascular death				
Coronary heart disease death	38	0.96 (0.72, 1.27)	0.84 (0.59, 1.20)	0.95 (0.73, 1.23)^§^
Cerebrovascular disease death	4	1.32 (0.73, 2.41)	1.13 (0.51, 2.51)	0.84 (0.39, 1.92)

All models were adjusted for age, race, sex, BMI, NDI, smoking, baseline co-morbidities, medications

^a^Estimated hazard ratios per increase of one standard deviation in the median long-term exposure to each traffic-related air pollutant

^b^Events include myocardial infarction, coronary revascularization, stroke, death from coronary heart disease, and death from cerebrovascular disease

^c^Events include myocardial infarction, coronary revascularization, and death from coronary heart disease

^d^Events include stroke and death from cerebrovascular disease

^§^*P*-value < 0.05 for interaction term, indicating statistically significant differences by elderly status in the association of the air pollutant with risk of the cardiovascular event

Table 4 shows the association between exposure to median long-term traffic-related air pollution and the risk of an incident cardiovascular event among diabetics and among non-diabetics. Among diabetics, an increase of one standard deviation in the exposure median long-term traffic-related air pollution was associated with an adjusted hazard ratio of 1.04 for NO_2_ (95% confidence interval [CI] 0.90 to 1.21), 1.06 for NO (95% CI 0.93 to 1.20), and 1.03 for BC (95% CI 0.89 to 1.18) for the time to the first cardiovascular event or death. Estimated effects were similar among diabetics and among non-diabetics, with no consistent pattern of increased risk among diabetics across the cardiovascular event endpoints. There were no statistically significant differences in the relationship between TRAP exposure and risk of CVD events by diabetes status.

**Table 4:** For diabetics and non-diabetics, risk of an incident cardiovascular event associated with each air pollutant

Outcome	Events	Hazard Ratio (95% CI)^a^		
		NO2	NO	Black carbon
Diabetics (*N* = 2898):				
First cardiovascular event				
Cardiovascular event or death^b^	216	1.04 (0.90,1.21)	1.06 (0.93,1.20)	1.03 (0.89,1.18)
Myocardial infarction	71	1.04 (0.81,1.33)	1.03 (0.83,1.29)	1.09 (0.86,1.37)
Revascularization	36	1.01 (0.70,1.46)	1.03 (0.75,1.42)	1.01 (0.72,1.41)
Stroke	102	1.09 (0.88,1.35)	1.08 (0.91,1.29)	1.04 (0.84,1.28)
Coronary heart disease event or death^c^	128	0.98 (0.81,1.20)	1.02 (0.86,1.22)	1.07 (0.90,1.28)
Cerebrovascular disease event or death^d^	103	1.09 (0.88,1.34)	1.07 (0.90,1.29)	1.01 (0.82,1.25)
Cardiovascular death				
Coronary heart disease death	47	0.91 (0.65,1.28)	1.00 (0.74,1.36)	1.17 (0.90,1.53)
Cerebrovascular disease death	9	1.12 (0.56,2.24)	0.94 (0.49,1.82)	0.90 (0.40,2.01)
Non-diabetics (*N* = 38,971):				
First cardiovascular event				
Cardiovascular event or death^b^	477	1.02 (0.93,1.13)	1.02 (0.93,1.12)	0.98 (0.89,1.07)
Myocardial infarction	147	1.10 (0.93,1.31)	1.10 (0.94,1.29)	1.03 (0.87,1.21)
Revascularization	94	0.99 (0.79,1.24)	1.00 (0.81,1.24)	1.04 (0.85,1.27)
Stroke	223	0.92 (0.78,1.07)	0.92 (0.79,1.08)	0.92 (0.80,1.06)
Coronary heart disease event or death^c^	266	1.08 (0.95,1.23)	1.07 (0.95,1.21)	1.04 (0.92,1.17)
Cerebrovascular disease event or death^d^	234	0.95 (0.81,1.10)	0.95 (0.82,1.10)	0.92 (0.81,1.06)
Cardiovascular death				
Coronary heart disease death	83	1.18 (0.94,1.48)	1.08 (0.87,1.33)	1.09 (0.88,1.35)
Cerebrovascular disease death	18	1.47 (0.95,2.28)	1.28 (0.88,1.87)	0.91 (0.53,1.58)

All models were adjusted for age, race, sex, BMI, NDI, smoking, baseline co-morbidities, medications

^a^Estimated hazard ratios per increase of one standard deviation in the median long-term exposure to each traffic-related air pollutant

^b^Events include myocardial infarction, coronary revascularization, stroke, death from coronary heart disease, and death from cerebrovascular disease

^c^Events include myocardial infarction, coronary revascularization, and death from coronary heart disease

^d^Events include stroke and death from cerebrovascular disease

The results of our sensitivity analyses are given in the Supplementary Material (Additional file 1). In our first sensitivity analysis, we fit the Cox PH models using age as time scale, with results given in Additional file 1: Table S1. We found that when using this alternative time scale, the point estimates and CIs estimated were extremely similar to those reported in our main analyses in Table 2. Additional file 1: Table S2 reports the estimated hazard ratios for the risk of an incident cardiovascular event associated with the TRAP exposures averaged using a 60 m buffer zone and a 120 m buffer zone, our second sensitivity analyses. Results were similar to those reported in main analyses in Table 2. In our third sensitivity analysis, we examined whether the relationship of TRAP to risk of CVD events varied by neighborhood. Results show that many of the association estimates appeared to be slightly stronger in East Oakland than in West/Downtown Oakland, although only revascularization showed statistically significant effect modification (Additional file 1: Table S3). We note, however, that there were a small number of revascularization events (*N* = 73 in East Oakland and *N* = 57 in Downtown/West Oakland).

## Discussion

This study exploring intra-urban variation in TRAP and incidence of CVD events, showed that street-level differences in long-term exposure to TRAP were associated with incidence of fatal and non-fatal cardiovascular disease events among the elderly. Though the effects among the general population of all adults were not statistically significant, the magnitudes of the effects were generally consistent with previous studies of air pollution and CVD events that used less spatially-resolved exposures over a larger region. This study represents an initial effort to leverage mobile monitoring measurements at the street level to understand how air pollution differences within small regions such between streets or blocks within urban neighborhoods may affect cardiovascular health. In contrast to studies that have assumed that people in one neighborhood share the same air pollution exposure, this study advances the field of TRAP research by demonstrating that street-level variation in TRAP exposure within urban neighborhoods contributes to differences in risk of cardiovascular events within small, localized populations.

The results of our main analyses studying all adults are consistent with previous studies of long-term exposure to traffic-related air pollution and outcomes of CVD events and CVD mortality. A meta-analysis of annual average NO_2_ concentrations and CVD mortality across twenty-two European cohorts reported an estimated HR of 1.01 (95% CI: 0.97 to 1.06) for total CVD mortality and 1.01 (95% CI: 0.93 to 1.10) for cerebrovascular mortality per 10 μg/m^3^ increase in NO_2_ [15]. The results of an analysis of 15 years of follow up in the California ACS CPSII study participants find a HR of 1.04 (95% CI: 0.99–1.09) for cardiovascular mortality associated with a 4.9 ppb increase in NO_2_ [16]. Cumulative NO_2_ exposure over decades was associated with a 12% increase in cardiovascular mortality for each increase of 5 parts per billion (HR 1.12 [95% CI: 1.07–1.17]) in a Canadian study of 205,440 adults [17]. Suggestive increases in CVD mortality have also been reported in relationship to long-term black smoke exposure (HR 1.04 [95% CI 0.95–1.13] per 10 μg/m^3^ increase) and traffic intensity on the nearest road (HR 1.05 [95% CI 0.99–1.12]) [18]. Our finding that that BC was associated with an increased risk of coronary heart disease death, but not with non-fatal incident CVD events, is consistent with the findings of a population-based cohort of 466,727 people living in Vancouver, where long term BC exposure was associated with a 6% higher risk of CVD mortality and 3% higher risk of CVD hospitalizations for every 0.8 μg/m^3^ change in exposure [5]. For myocardial infarction, long-term NO_2_ exposure has been associated with an estimated HR of 1.08 (95% CI: 1.03 to 1.12) per IQR increase in 10-year mean NO_2_ exposure [19] and a HR of 0.98 (95% CI: 0.93 to 1.03) per IQR increase (10.7 μg/m^3^) in annual mean NO_2_ exposure [12]. For incidence of coronary events, an increase of 10 μg/m^3^ in annual mean NO_2_ exposure has been associated with an estimated HR of 1.03 (95% CI: 0.97 to 1.08) [20] and 1.03 (95% CI: 1.00 to 1.07) [3]. For incident stroke, long-term exposure to NO_2_ over several decades was associated with a 5% increased risk per interquartile range increase (HR 1.05 [95% CI 0.99–1.11]) [21]. These studies mainly utilized LURs or dispersion modelling for exposure assessment, encompassed much geographically much larger areas (entire countries, states or cities) and large populations followed over extended periods of time. In contrast we are using mobile monitoring to measure NO_2_ concentrations at a much finer resolution in a very small geographic area with a follow-up time of up to 6 years. Although the magnitude of the point estimates and CIs are not directly comparable between our study and these previous studies because the exposure metrics vary in their characterization of long-term TRAP exposure, our results broadly appear to be consistent with previous studies.

Our results for NO2, NO and BC did not always show the same pattern of associations across the CVD health outcomes. Though overall NO, NO2 and BC are all types of traffic-related air pollution, we found moderate correlation between BC and NO/NO2 and different patterns of CVD health effects for the pollutants. These differences may reflect the different traffic sources of BC (diesel emissions) and NO2/NO (all vehicular emissions). Furthermore, the differences in spatial patterns of TRAP found in this study may reflect in part the impact of local traffic management decisions (e.g. designated truck routes and areas where trucks are prohibited). In large cities, road traffic is a main source of NO_2_ and a major contributor to PM emissions. For example, a study of Paris found that road traffic accounted for 60% of emissions of nitrogen oxides and 30% of PM emissions [22]. Furthermore, NO_2_ and PM2.5 may be highly correlated in some cities; modeled exposures of NO2 and PM2.5 for two cohorts in London had a reported correlation of 0.83 [23]. While nitrogen oxides, black carbon, and traffic measures have been linked to cardiovascular events such as myocardial infarction, stroke, heart failure and cardiovascular mortality, the evidence for an association between PM_2.5_ exposure and CVD far exceeds that for other components [24]. The American Heart Association (AHA) Scientific Statement on Particulate Matter Air Pollution and Cardiovascular Disease concludes that “the overall evidence is consistent with a causal relationship between PM_2.5_ exposure and cardiovascular morbidity and mortality,” [25] while in contrast, the relationship between long-term NO_2_ exposure and cardiovascular health effects has been deemed only suggestive of a causal relationship by the US Environment Protection Agency [4]. Although few studies have examined the relationship of long-term NO to CVD endpoints, a previous study reported that NO_2_ and NO had similar magnitudes of association with CHD mortality [5]. Further research is needed to understand the differences in effects between components of TRAP as well as to understand whether TRAP pollutants are also causally related to cardiovascular health effects.

### Susceptible populations

Our study found that the elderly are more susceptible than non-elderly adults to the effects of TRAP. In previous research, the elderly have been considered a sensitive subpopulation to the effects of air pollution because of the established association between air pollution and mortality endpoints. Some studies of air pollution and CVD events only include older subjects, such as studies of the Medicare population [26, 27]; these studies are able to establish associations of air pollution on risk of CVD events among the elderly but cannot address whether the elderly are indeed more susceptible than the non-elderly because there is no non-elderly comparison group. However, evidence is mixed on whether there is effect modification for older ages in the effects of air pollution on risk of CVD events. Some studies of both long-term and short-term air pollution exposure and CVD mortality have reported effects that appear stronger in the elderly with suggestive evidence of effect modification that did not meet the threshold of statistical significance [20, 28]. In addition, some studies of long-term air pollution exposure and CVD mortality have found no evidence of effect modification by older age [15]. The stronger associations we observed among the elderly that represented statistically significant effect modification add to a growing body of evidence suggesting that elderly populations may have increased susceptibility to air pollution. We note that we performed 24 statistical tests for interaction by elderly age and 10 of them were statistically significant at level 0.05.

Our study did not find differences between diabetic and non-diabetic subjects in susceptibility to the effects of air pollution. Previous studies of air pollution and cardiovascular health suggest diabetic individuals may experience a heightened risk to the effects of air pollution exposure [29, 30]. Diabetes has been examined as a potential effect modifier in previous studies of TRAP exposure with cardiovascular outcomes including heart rate variability, ventricular arrhythmias, blood pressure, hospital admissions and ED visits for IHD and MI, but the evidence has been inconsistent as to whether diabetes modifies TRAP-related cardiovascular effects [31–34]. Studies of long-term NO_2_ exposure with stroke and with total mortality did not find evidence of effect modification by diabetes [21, 35], while a study of short-term NO_2_ exposure with total mortality did find that subjects with diabetes experienced a stronger susceptibility to the effects of NO_2_ [36]. We found no evidence of effect modification by diabetes in our study. We did not have sufficient statistical power to look specifically at whether diabetes may influence susceptibility among the elderly only, which would have required a three-way interaction term.

### Mechanisms

Several recent review papers have summarized the hypothesized mechanisms that underlie the relationship between air pollution and CVD [22, 24]. Air pollution exposure, including diesel exposure, has been shown to induce oxidative stress and endothelial dysfunction, which is followed by alteration of circulating lipids [22]. Some evidence indicates that respiratory irritation in response to NO or NO_2_ exposure leads to activation of the autonomic nervous system, generation of vasoactive molecules and reactive oxidant species entering the systemic circulation, which in turn cause endothelial dysfunction, systemic inflammation and altered heart rhythm [4]. All these are important biological pathways in the pathophysiology of cardiovascular disease. Evidence from the Normative Aging Study has shown that short and long term BC exposures are associated with several subclinical cardiovascular health endpoints, including Heart Rate Variability [37], carotid intima-media thickness [38], systolic and diastolic blood pressure [39], circulating biomarkers of inflammation and endothelial response [40], suggesting that inflammation, endothelial dysfunction and the autonomic nervous system play a role in the etiology of its effects on CVD. In addition, traffic intensity, a measure of long-term exposure to traffic-related air pollution, has been associated carotid intima-media thickness [41]. However, toxicological and controlled human chamber studies have not been able to tease apart the effects of PM and BC [42].

### Strengths and limitations

This study has a number of strengths and weaknesses. A key innovation of our study is the use of an intensive mobile monitoring campaign to obtain TRAP measurements at 30 m resolution on every street in the Oakland neighborhoods where the study population lived. This measurement approach captured the variability of these pollutants at a higher resolution than has been possible in previous studies, enabling us to better examine differences in TRAP in the urban environment. We expect that our high-resolution measurements provided a more spatially resolved map with lower measurement error than previous studies using only land-use regression. Land-use regression is thought to induce mainly Berkson error [43, 44], which does not induce bias in effect estimates but does reduce precision, making effects more difficult to detect. We expect that the main advantage in using mobile monitors as compared to land-use regression models would be a reduction in Berkson error and a resulting increase in precision. This increased precision allows us to study differences at smaller neighborhood scales than can be studied using existing land use regression methods. There is also a classical measurement error component in land-use regression models [44], which is thought to bias effects toward the null. Certain cases of spatial measurement error due to model misspecification may lead to bias away from the null [45]. In addition, all studies that examine exposures at residential addresses have a component of measurement error due to limited information on time-activity patterns [46].

Our study assumed spatial stability in the median air pollution exposure patterns over the follow-up period. Although we could have built our study cohort with a start date prior to 2010, we wanted to balance the need for having a long enough follow-up time to observe events while also upholding the assumption of spatial stability in the TRAP exposure patterns over the follow-up. A period of 6 years fell within the range of previous studies of long-term air pollution effects. For example, Miller et al. 2007 followed subjects from 1994 to 2002 with air pollution measured during the year 2000 [2] and Hart et al. 2013 followed subjects from 1990 to 2008 and used modeled NO_2_ exposures for the year 2000 [47]. Furthermore, a previous study of NO_2_ over 20 years found that the spatial distributions of NO2 did not change appreciably over the follow-up period [8]. TRAP measurements in this study were only taken during the daytime and on weekdays, and only during 1 year; thus these median air pollution exposure patterns may not represent the patterns at night or on weekends, and do not reflect any changes in the overall pattern from year to year. We represented long-term exposures by calculating the median over repeated mobile measurements at each location, which was found to be spatially stable as the number of repeated measurements increased [7]. The typical exposure used in studies of long-term effects of air pollution is the one-year or multi-year mean of the 24-h average exposure, although most etiologically relevant metric of long-term exposure to TRAP with respect to long-term risk of CVD events has not been established.

The study cohort members were all members of Kaiser Permanente Northern California who had equal access to health care, which limits confounding by health care access and increases the internal validity of our study. We controlled for a number of key confounders, however there still may be residual confounding in some variables such as SES, which was controlled for by area-level SES measures rather than individual-level SES, and smoking, which was controlled for by smoking status but not by packyears or number of year smoked. The average duration of follow-up was 2.5 years, which may be too short to fully capture the association between long-term exposure to TRAP and time to CVD event. The generalizability of our study is limited because the small geographic region which includes subjects with lower SES on average than the general KPNC population and is comprised of a high proportion of African Americans. Our exclusion criteria are consistent with previous studies that limit the study population to subjects without history of CVD, focusing on incident cardiovascular events in relation to long-term air pollution exposure [2, 48, 49]. We excluded subjects with CHF who are typically analyzed as a separate group in air pollution studies to examine recurrent CVD events [50–52] since those subjects experience much higher rates of CVD events than the general population and may be more susceptible to cardiac effects of air pollution than the general population. In addition, we are capturing only the insured population with access to health care within that region.

We controlled for hypertension as a comorbidity as well as hypertension medication use at baseline to account for the greater risk of CVD events among subjects whose hypertension is not controlled by medication as compared to those with hypertension whose blood pressure is controlled by medication. Approximately 70% of subjects with hypertension were taking hypertensive medications (Table 1). The ability to adjust for the effects of hypertension on the risk of CVD events at this level of detail is an advantage of our study over many previous studies that do not have such detailed medical record and pharmacy data available. All covariates were included as baseline covariates in the regression models. Notably, recent studies and a recent meta-analysis have found that air pollution exposures may contribute to the incidence of hypertension [53]. Thus, our rationale for not adjusting for changes in comorbidity status and medication use during follow-up was that worsening disease status during follow-up may reflect a mediating pathway between air pollution exposure and cardiovascular events.

Our main analyses used time on study as the time scale for the Cox PH model, adjusting for baseline age as a model covariate. Time on study (i.e. follow-up time) has often been used as the time scale for the Cox PH model, although interest in alternative time scales such as age as the time scale has grown with the increase in large scale epidemiologic cohort studies [54]. When using time on study as the time scale, an assumption is needed that the baseline hazard is an exponential function of age to ensure unbiased estimation of parameters representing covariate effects. When this assumption is met, then either time scale will yield unbiased estimates of covariate relative hazards [55]. However, violation of this assumption combined with substantial correlation between the exposure of interest and age can results in substantial bias in parameter estimates [55]. The main advantage of using age as the time scale is flexible control for age effects where any non-linear effects of age are handled implicitly; this avoids the need to directly model the effect of age that satisfies the proportional hazard assumption underlying the Cox model [54]. In this study, we found no violation of assumptions in the distribution of age in the hazard function. We further conducted a sensitivity analysis using age as time scale, and we found that point estimates and CIs were extremely similar for either time scale (Additional file 1: Table S2, Supplementary Material).

One limitation of this study was low power to detect small effect sizes. Although we were able to capture fine-scale differences in exposure to TRAP within neighborhoods, we were limited by the number of events in our study due to small the geographic coverage of our study area, and the duration of follow up. Further, the small geographic region restricted the range in exposures, since many people lived on streets with similar levels of exposure with fewer people living in areas of high exposure. All of the above reduced the power of the analyses. It is plausible that in a larger study we would be able to detect statistically significant associations in the general population, and effect modification by other susceptibility factors.

Using mobile monitoring measurements of NO_2_, NO, and BC collected at 30 m resolution on each street represents an innovation in the study of TRAP and cardiovascular health. While many previous studies have assigned the same air pollution exposure to people in one neighborhood, this study moves the field of TRAP research forward by demonstrating that street-level variation in TRAP exposure within urban neighborhoods may contribute to increased risk of cardiovascular events. The results of this study suggest that mobile monitoring measurements of TRAP may be useful in studying the relationship of intra-urban differences in air pollution exposure and risk of CVD. Future studies of TRAP using spatially-resolved mobile monitoring that cover larger areas and assess exposures at multiple times of day throughout the year would be beneficial to our understanding of the long-term health effects related to intra-urban differences in TRAP.

## Conclusions

The results of this study show that higher intra-urban TRAP exposures are associated with a higher risk of CVD events among the elderly. In addition, our results in the full study cohort are broadly consistent with the associations found in other studies that encompass a larger geographic region with less spatially-resolved exposures. Thus, this study suggests that intra-urban differences in TRAP exposures contribute to differences in cardiovascular health within neighborhoods. These results have implications for management of local sources, zoning, urban development and transportation planning. Further studies of fine-scale differences in TRAP that address the limitations of our study are needed to confirm our findings.

## Additional file

Additional file 1: **Table S1.** Risk of an incident cardiovascular event associated with each air pollutant, hazard ratios estimated in a Cox model using age as time scale. **Table S2.** Estimated hazard ratios for the risk of an incident cardiovascular event associated with an increase of one standard deviation in the exposure to median long-term traffic-related air pollution averaged using a 60m buffer zone and a 120m buffer zone. **Table S3.** Estimated hazard ratios for the risk of an incident cardiovascular event associated with an increase of one standard deviation in the exposure to median long-term traffic-related air pollution in East Oakland and in Downtown/West Oakland. **Figure S1.** Study region in Oakland with black dots indicating the locations where air pollution exposures were measured in the areas of West/Downtown Oakland and East Oakland. **Figure S2.** Correlations between median long-term street-level exposures to traffic-related air pollution at study cohort residential address locations in Oakland. (DOCX 898 kb)

## Abbreviations

BC: Black carbon
CI: Confidence interval
CVD: Cardiovascular disease
IQR: Inter-quartile range
KPNC: Kaiser Permanente Northern California
MI: Myocardial infarction
NDI: Neighborhood deprivation index
NO: Nitric oxide
NO_2_: Nitrogen dioxide
PH: Proportional-hazards
PM_2.5_: Particulate matter less than 2.5 μm in diameter
SD: Standard deviation
SES: Socioeconomic status
TRAP: Traffic related air pollutants
