# Supplementary material for: High-resolution mapping of traffic related air pollution with Google street view cars and incidence of cardiovascular events within neighborhoods in Oakland, CA
Source: Environ Health. 2018 May 15;17:38. doi: 10.1186/s12940-018-0382-1 (PMC5952592; doi:10.1186/s12940-018-0382-1)
Supplement: Supplementary file 1 — Table S1. Risk of an incident cardiovascular event associated with each air pollutant, hazard ratios estimated in a Cox model using age as time scale. Table S2. Estimated hazard ratios for the risk of an incident cardiovascular event associated with an increase of one standard deviation in the exposure to median long-term traffic-related air pollution averaged using a 60m buffer zone and a 120m buffer zone. Table S3. Estimated hazard ratios for the risk of an incident cardiovascular event associated with an increase of one standard deviation in the exposure to median long-term traffic-related air pollution in East Oakland and in Downtown/West Oakland. Figure S1. Study region in Oakland with black dots indicating the locations where air pollution exposures were measured in the areas of West/Downtown Oakland and East Oakland. Figure S2. Correlations between median long-term street-level exposures to traffic-related air pollution at study cohort residential address locations in Oakland. (DOCX 898 kb) [file 12940_2018_382_MOESM1_ESM.docx]

**Supplementary Materials.**

**High-Resolution Mapping of Traffic Related Air Pollution with Google Street View Cars and Incidence of Cardiovascular Events within Neighborhoods in Oakland, CA**

Stacey E. Alexeeff, Ananya Roy, Jun Shan, Xi Liu, Kyle Messier, Josh Apte, Christopher Portier, Stephen Sidney, Stephen K. Van Den Eeden

**Table S1.** Risk of an incident cardiovascular event associated with each air pollutant, hazard ratios estimated in a Cox model using age as time scale.

| **Outcome** | **Events** | **Hazard Ratio (95% CI)** | | |
| --- | --- | --- | --- | --- |
|  |  | NO2 | NO | Black carbon |
| **First cardiovascular event** |  |  |  |  |
| Cardiovascular event or death* | 693 | 1.03 (0.95,1.13) | 1.06 (0.98,1.14) | 1.00 (0.93,1.09) |
| Myocardial infarction | 224 | 1.07 (0.92,1.25) | 1.07 (0.94,1.22) | 1.03 (0.90,1.19) |
| Revascularization | 130 | 0.95 (0.77,1.16) | 0.98 (0.81,1.19) | 0.99 (0.83,1.19) |
| Stroke | 325 | 1.00 (0.88,1.13) | 1.02 (0.91,1.15) | 0.99 (0.88,1.11) |
| Coronary heart disease event or death^†^ | 394 | 1.05 (0.93,1.17) | 1.06 (0.96,1.18) | 1.04 (0.93,1.15) |
| Cerebrovascular disease event or death^‡^ | 337 | 1.01 (0.89,1.14) | 1.03 (0.92,1.15) | 0.99 (0.88,1.11) |
| **Cardiovascular death** |  |  |  |  |
| Coronary heart disease death | 130 | 1.12 (0.92,1.36) | 1.11 (0.94,1.30) | 1.10 (0.92,1.31) |
| Cerebrovascular disease death | 27 | 1.38 (0.89,2.13) | 1.12 (0.82,1.55) | 1.01 (0.65,1.59) |

* Events include myocardial infarction, coronary revascularization, stroke, death from coronary heart disease, and death from cerebrovascular disease.

^†^ Events include myocardial infarction, coronary revascularization, and death from coronary heart disease.

^‡^ Events include stroke and death from cerebrovascular disease

**Table S2.** Estimated hazard ratios for the risk of an incident cardiovascular event associated with an increase of one standard deviation in the exposure to median long-term traffic-related air pollution averaged using a 60m buffer zone and a 120m buffer zone.

| **Outcome** | **Events** | **Hazard Ratio (95% CI)** | | |
| --- | --- | --- | --- | --- |
|  |  | NO2 | NO | Black carbon |
| 60m buffer zone: |  |  |  |  |
| **First cardiovascular event** |  |  |  |  |
| Cardiovascular event or death* | 693 | 1.03 (0.95,1.13) | 1.06 (0.98,1.14) | 1.00 (0.93,1.09) |
| Myocardial infarction | 224 | 1.07 (0.92,1.25) | 1.07 (0.94,1.22) | 1.03 (0.90,1.19) |
| Revascularization | 130 | 0.95 (0.77,1.16) | 0.98 (0.81,1.19) | 0.99 (0.83,1.19) |
| Stroke | 325 | 1.00 (0.88,1.13) | 1.02 (0.91,1.15) | 0.99 (0.88,1.11) |
| Coronary heart disease event or death^†^ | 394 | 1.05 (0.93,1.17) | 1.06 (0.96,1.18) | 1.04 (0.93,1.15) |
| Cerebrovascular disease event or death^‡^ | 337 | 1.01 (0.89,1.14) | 1.03 (0.92,1.15) | 0.99 (0.88,1.11) |
| **Cardiovascular death** |  |  |  |  |
| Coronary heart disease death | 130 | 1.12 (0.92,1.36) | 1.11 (0.94,1.30) | 1.10 (0.92,1.31) |
| Cerebrovascular disease death | 27 | 1.38 (0.89,2.13) | 1.12 (0.82,1.55) | 1.01 (0.65,1.59) |
| 120m buffer zone: |  |  |  |  |
| **First cardiovascular event** |  |  |  |  |
| Cardiovascular event or death* | 693 | 1.03 (0.95,1.13) | 1.05 (0.97,1.13) | 1.03 (0.95,1.11) |
| Myocardial infarction | 224 | 1.10 (0.95,1.27) | 1.06 (0.93,1.21) | 1.05 (0.91,1.20) |
| Revascularization | 130 | 0.98 (0.80,1.20) | 1.00 (0.83,1.21) | 1.00 (0.83,1.20) |
| Stroke | 325 | 0.99 (0.86,1.12) | 1.01 (0.90,1.14) | 1.01 (0.90,1.14) |
| Coronary heart disease event or death^†^ | 394 | 1.06 (0.94,1.19) | 1.05 (0.95,1.16) | 1.05 (0.94,1.16) |
| Cerebrovascular disease event or death^‡^ | 337 | 1.00 (0.88,1.13) | 1.02 (0.91,1.14) | 1.01 (0.90,1.14) |
| **Cardiovascular death** |  |  |  |  |
| Coronary heart disease death | 130 | 1.10 (0.91,1.34) | 1.06 (0.90,1.24) | 1.07 (0.89,1.27) |
| Cerebrovascular disease death | 27 | 1.27 (0.82,1.98) | 1.06 (0.73,1.54) | 1.00 (0.63,1.57) |

* Events include myocardial infarction, coronary revascularization, stroke, death from coronary heart disease, and death from cerebrovascular disease.

^†^ Events include myocardial infarction, coronary revascularization, and death from coronary heart disease.

^‡^ Events include stroke and death from cerebrovascular disease

**Table S3.** Estimated hazard ratios for the risk of an incident cardiovascular event associated with an increase of one standard deviation in the exposure to median long-term traffic-related air pollution in East Oakland and in Downtown/West Oakland.

| **Outcome** | **Events** | **Hazard Ratio (95% CI)** | | |
| --- | --- | --- | --- | --- |
|  |  | NO2 | NO | Black carbon |
| East Oakland: |  |  |  |  |
| **First cardiovascular event** |  |  |  |  |
| Cardiovascular event or death* | 389 | 1.11 (0.95,1.30) | 1.11 (0.97,1.26) | 0.98 (0.87,1.10) |
| Myocardial infarction | 133 | 1.23 (0.95,1.58) | 1.17 (0.96,1.43) | 1.05 (0.87,1.28) |
| Revascularization | 73 | 1.47 (1.08,2.00) ^§^ | 1.30 (1.01,1.66) ^§^ | 1.05 (0.81,1.36) |
| Stroke | 195 | 1.04 (0.83,1.30) | 1.02 (0.84,1.24) | 0.98 (0.83,1.15) |
| Coronary heart disease event or death^†^ | 213 | 1.18 (0.96,1.45) | 1.18 (1.00,1.39) | 1.03 (0.88,1.20) |
| Cerebrovascular disease event or death^‡^ | 201 | 1.03 (0.82,1.28) | 1.01 (0.84,1.23) | 0.96 (0.82,1.13) |
| **Cardiovascular death** |  |  |  |  |
| Coronary heart disease death | 61 | 0.88 (0.55,1.40) | 1.00 (0.66,1.52) | 0.99 (0.74,1.32) |
| Cerebrovascular disease death | 12 | 0.62 (0.22,1.75) | 0.49 (0.11,2.11) | 0.81 (0.43,1.52) |
| Downtown/West Oakland: |  |  |  |  |
| **First cardiovascular event** |  |  |  |  |
| Cardiovascular event or death* | 304 | 0.96 (0.82,1.12) | 0.98 (0.87,1.11) | 1.00 (0.87,1.14) |
| Myocardial infarction | 91 | 0.98 (0.75,1.29) | 1.00 (0.82,1.23) | 1.05 (0.83,1.33) |
| Revascularization | 57 | 0.69 (0.47,1.02) ^§^ | 0.83 (0.60,1.14) ^§^ | 1.05 (0.79,1.40) |
| Stroke | 130 | 0.96 (0.76,1.21) | 0.99 (0.82,1.19) | 0.96 (0.77,1.19) |
| Coronary heart disease event or death^†^ | 181 | 0.92 (0.76,1.13) | 0.95 (0.81,1.11) | 1.05 (0.89,1.23) |
| Cerebrovascular disease event or death^‡^ | 136 | 1.00 (0.80,1.25) | 1.02 (0.86,1.21) | 0.96 (0.77,1.18) |
| **Cardiovascular death** |  |  |  |  |
| Coronary heart disease death | 69 | 1.03 (0.74,1.42) | 0.97 (0.75,1.26) | 1.10 (0.85,1.43) |
| Cerebrovascular disease death | 15 | 2.03 (1.18,3.52) | 1.27 (0.89,1.80) | 1.00 (0.43,2.31) |

* Events include myocardial infarction, coronary revascularization, stroke, death from coronary heart disease, and death from cerebrovascular disease.

^†^ Events include myocardial infarction, coronary revascularization, and death from coronary heart disease.

^‡^ Events include stroke and death from cerebrovascular disease

^§^ P-value < 0.05 for interaction term, indicating statistically significant differences by neighborhood in the association of the air pollutant with risk of the cardiovascular event

**Figure S1.** Study region in Oakland with black dots indicating the locations where air pollution exposures were measured in the areas of West/Downtown Oakland and East Oakland.

**
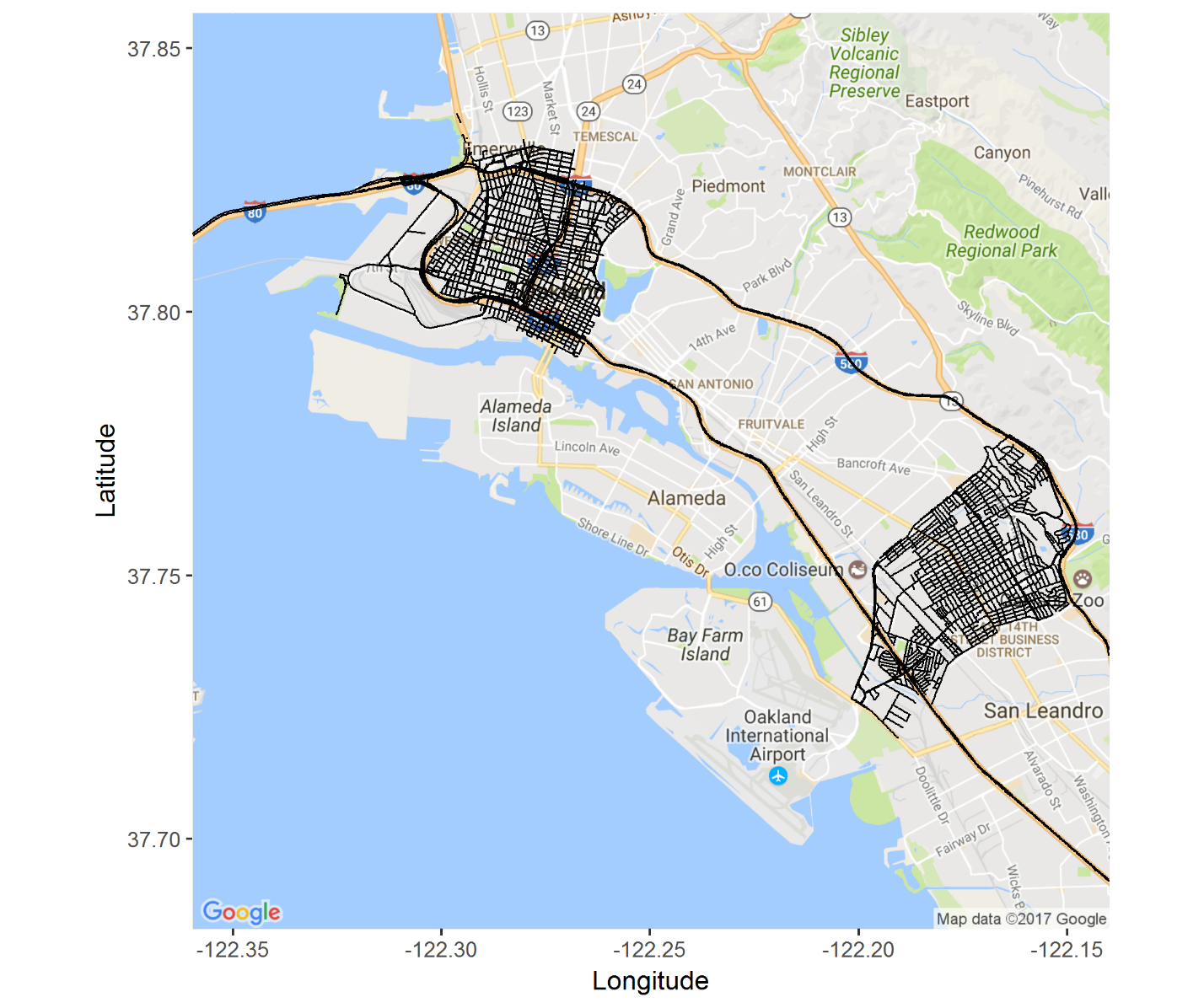
**

**Figure S2.** Correlations between median long-term street-level exposures to traffic-related air pollution at study cohort residential address locations in Oakland.

**
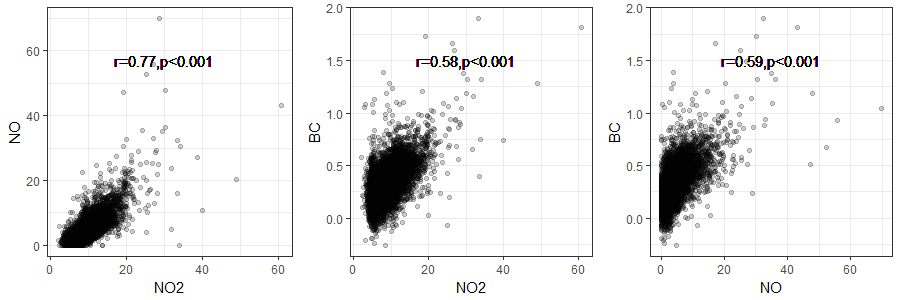
**
